# 4-octyl itaconate modulates virulence-associated phenotypes and oxidative stress resistance in avian pathogenic *Escherichia coli* by targeting *menB* and *wza*

**DOI:** 10.1016/j.psj.2025.106202

**Published:** 2025-12-06

**Authors:** Chuanyan Che, Xiuqi Fan, Haiyang Wang, Zhihao Wang, Yinli Bao, Wei Jiang, Saqib Nawaz, Huifang Yin, Xiangan Han

**Affiliations:** a College of Animal Science, Anhui Science and Technology University, Chuzhou, 233100, China; b Shanghai Veterinary Research Institute, the Chinese Academy of Agricultural Sciences (CAAS), 518 Ziyue Road, Shanghai 200241, China; c Anhui Province Key Laboratory of Animal Nutritional Regulation and Health, Chuzhou, 233100, China; d Engineering Research Center for the Prevention and Control of Animal Original Zoonosis, Fujian Province, College of Life Science, Longyan University, Longyan 364012, China; e Anhui Province Key Laboratory of Veterinary Pathobiology and Disease Control, College of Veterinary Medicine, Anhui Agricultural University, Hefei 230036, PR China

**Keywords:** Avian Pathogenic *Escherichia coli* (APEC), 4-octyl itaconate, Biological characteristics, Transcriptomics

## Abstract

Avian Pathogenic *Escherichia coli* (APEC) causes substantial economic losses to the poultry industry, primarily due to its diverse serotypes and extensive drug resistance. Itaconate, a metabolite of the tricarboxylic acid (TCA) cycle, demonstrates antimicrobial activity. However, the effect of itaconate on the biological characteristics of APEC remains unexplored. Because itaconate itself has poor membrane permeability, its cell-permeable derivative, 4-OI, has emerged as a promising alternative and has attracted significant attention. This study investigates the impact of 4-OI on the biological properties of APEC, including growth, biofilm formation, antioxidant stress response, and acid-base tolerance. The results revealed that the addition of 200 μM or 400 μM 4-OI to APEC94 cultures for 14 h significantly inhibited bacterial growth. Specifically, 4-OI at concentrations of 100 μM to 400 μM markedly suppressed biofilm formation in APEC. Conversely, concentrations between 50 μM and 400 μM significantly enhanced APEC94 survival under H₂O₂-induced oxidative stress. Transcriptomic analysis demonstrated that the exogenous application of 200 μM 4-OI significantly altered the transcription of the *wza* and *menB* genes. Further investigation demonstrated that 4-OI targets the *wza* and *menB* genes, potentiating its inhibitory effect on biofilm formation and mitigating H₂O₂-induced suppression in APEC94. Furthermore, treatment with 200 μM 4-OI reduced bacterial tolerance against acid-base stress and osmotic pressure, an effect that was contingent upon the presence of the *wza* and *menB* genes. In conclusion, this study reveals the multifaceted effects of 4-OI on APEC, including its inhibitory effects on growth, biofilm formation, oxidative stress response, and tolerance to acid-base and osmotic stress. Furthermore, it demonstrates that the *wza* and *menB* genes mediate the enhancement of oxidative stress resistance in APEC induced by 4-OI.

## Introduction

Colibacillosis caused by Avian Pathogenic *Escherichia coli* (APEC) is a significant bacterial disease in poultry worldwide (Helmy et al., 2018). Clinically, it manifests as systemic inflammatory responses such as pericarditis, airsacculitis, and perihepatitis, and is associated with elevated morbidity and mortality rates, thereby causing substantial economic losses to the poultry industry (Shen, et al., 2024). APEC harbors and employs various virulence factors and phenotypic traits, including growth characteristics, biofilm formation, resistance to oxidative stress, acid-base, and osmotic stress tolerances, to cause colibacillosis in poultry (Kathayat, et al., 2021). These factors contribute to various pathogenic processes during APEC infection, including adherence to host cells, invasion, survival within phagocytes, and tissue colonization (Guerra, et al., 2018; Li, et al., 2020; Sarowska, et al., 2019). Therefore, investigating these factors is crucial for making novel and effective strategies for the prevention and control of APEC infections.

Itaconate, chemically designated as 2-methylsuccinic acid, was first isolated in 1836 by the Swiss chemist Samuel Baup. Hans Krebs described itaconate in his laboratory notebooks, along with succinate, malate, and other key metabolites, while working to elucidate the intermediates of the tricarboxylic acid (TCA) cycle (Holmes, 1980). Much of the research on itaconate involves the use of its derivative, 4-octyl itaconate (4-OI), which exhibits enhanced cellular permeability relative to itaconate. Moreover, 4-OI exhibits comparable thiol reactivity with other derivatives and is metabolized intracellularly to itaconate (Runtsch, et al., 2022). In recent years, a growing body of experimental evidence has indicated that itaconate exhibits both antibacterial and immunomodulatory activities (Peace and O'Neill, 2022). A key antibacterial mechanism involves inhibition of bacterial isocitrate lyase (ICL) (McFadden and Purohit, 1977; Nguyen, et al., 2019). ICL is a bacterial enzyme critical for the glyoxylate shunt—a metabolic pathway essential during infection; hence, inhibition of ICL by itaconate restricts the growth of pathogens that depend on ICL activity (Lorenz and Fink, 2002; Muñoz-Elías and McKinney, 2005). Itaconyl-CoA, a metabolite of itaconate, also acts as an inhibitor of methylmalonyl-CoA mutase (MCM) in bacteria such as *Mycobacterium tuberculosis*, thereby disrupting propionyl-CoA–dependent bacterial growth (Ruetz, et al., 2019).

However, the effect of exogenous itaconate on APEC remains to be elucidated. Therefore, this study investigates the impact of different concentrations of 4-OI on the phenotypic traits of APEC. To elucidate the mechanisms underlying 4-OI action on APEC, we performed transcriptomic sequencing and validation. Gene knockout experiments were subsequently performed to assess the role of target genes in mediating 4-OI effects. Our findings advance understanding of the pathogenic mechanisms of APEC and provide insights for the development of novel antibacterial agents, which is especially critical given the growing challenge of antimicrobial resistance. This study presents a conceptual framework for developing new antimicrobial strategies to control APEC. We hypothesized that 4-OI would significantly alter key biological characteristics of APEC and that these effects would be mediated through specific genetic targets.

## Materials and methods

### Bacterial strains and plasmids

The clinically isolated APEC O78 serotype strain APEC94, along with the plasmids pCas and pTarget used for gene knockout, were maintained in our laboratory. All strains were grown in Luria-Bertani (LB) broth at 37°C. 4-OI (Hongye Biotechnology, Shanghai, China) was added at concentrations of 50, 100, 200, and 400 μM when required.

### Measurement of bacterial growth

Each strain was cultured in LB liquid medium until reaching the mid-log phase, and then subcultured at a ratio of 1:100 into flasks containing 100 mL of LB broth supplemented with 4-OI at final concentrations of 0, 50, 100, 200, and 400 μM. The cultures were incubated at 37°C with shaking at 180 rpm. The optical density at 600 nm (OD₆₀₀) was measured every 2 h using a spectrophotometer (Bio-Rad, Hercules, CA, USA). Bacterial growth curves were generated from the data.

### Biofilm formation assay

A volume of 150 µL of LB broth containing 200 µM 4-OI was added to each well of a 96-well plate. Then, 15 µL of bacterial suspension was inoculated into each well, and the plate was incubated statically at 25 °C for 16 h, as this temperature optimizes and standardizes biofilm production under laboratory conditions (Nawaz, et al., 2025). After incubation, the wells were gently washed three times with PBS and dried at 60 °C to remove residual liquid. Next, 200 µL of a 0.1 % (w/v) crystal violet solution (Shaoxin Biotechnology, Shanghai, China) was added to each well, and the plate was incubated at 37 °C for 20 min. The crystal violet was then removed, and the wells were rinsed three times with PBS and air-dried at room temperature. Subsequently, 200 µL of 95 % ethanol was added to each well to dissolve the stained biofilm, followed by incubation at 37 °C for 20 min. The absorbance at 595 nm (OD₅₉₅) was measured using a microplate reader (Thermo Fisher Scientific, Waltham, MA, USA). The results were recorded and analyzed.

### Assessment of oxidative stress tolerance

Three milliliters of LB medium and 2 μL of 6.5 mM H₂O₂ were added to sterile test tubes, to which 200 μL of bacterial suspension (OD₆₀₀ = 1.0) was added. For the experimental group, 60 μL of 10 mM 4-OI (final concentration 200 μM) was added, while the control group received no 4-OI. Three replicates were prepared for each group. The tubes were incubated at 37 °C with shake-flask culture at 180 rpm for 1 h. After treatment, the bacterial cultures were serially diluted with sterile PBS. Subsequently, 3 μL of each dilution was spotted onto LB agar plates, and 20 μL of the gradient dilutions was added to each well. The plates were incubated at 37 °C for 12 h, and the number of colony-forming units (CFU) was enumerated.

### Determination of acid-base tolerance

A 100 μL aliquot of bacterial suspension (OD₆₀₀ = 1.0) was inoculated into 5 mL of LB medium adjusted to pH values of 4, 5, 6, 7, 8, 9, and 10, respectively. For the experimental groups, 100 μL of 10 mM 4-OI solution was added to the medium at each pH level (resulting in a final concentration of 200 μM), while other conditions remained consistent with the control group. Each pH gradient was tested with three replicates. The tubes were incubated at 37 °C with shake-flask culture at 180 rpm for 12 h, after which the optical density at 600 nm (OD₆₀₀) was measured.

### Determination of osmotic stress tolerance

Bacterial cultures were grown to mid-log phase (OD₆₀₀ = 1.0. For the control group, 200 µL of culture was inoculated into 3 mL of LB broth supplemented with 0.5 %, 1 %, 2 %, or 4 % (w/v) NaCl. For the experimental group, 4-OI was added to a final concentration of 200 µM, while all other conditions remained identical to the control. Each concentration gradient was tested in triplicate using sterile glass test tubes. The tubes were incubated at 37 °C with shake-flask culture at 180 rpm for 1 h. After incubation, the cultures were serially diluted 10-fold with sterile PBS. A 20 µL aliquot of each diluted suspension was evenly spread onto LB agar plates to facilitate the formation of distinct single colonies. The plates were then incubated at 37 °C for 12 h, after which the number of single colonies was counted. The CFU were calculated, and the results were analyzed.

### Transcriptome sequencing and analysis

The APEC94 strain was cultured at 37°C until the optical density at 600 nm (OD₆₀₀) reached 1.0. Two sterile conical flasks, each containing 100 mL of LB broth, were prepared; one flask was supplemented with 4-OI to a final concentration of 200 μM. Then, 100 μL of the APEC94 bacterial culture was inoculated into each flask and incubated at 37°C until the OD₆₀₀ reached 1.0 again. A 6 mL aliquot of each culture was collected into a 5 mL EP tube, and the bacterial cells were washed three times with sterile PBS, resuspended in PBS, and finally centrifuged and stored in cryotubes. The samples were sent to Biotechnology Corporation (Shanghai, China) for transcriptome sequencing. Differentially expressed genes (DEGs) between the sample groups were identified using the edgeR package. The resulting p-values were adjusted for multiple hypothesis testing. Fold-change values were calculated based on FPKM to quantify the magnitude of differential expression. The identified DEGs were mapped to various entries in functional databases, and the number of genes enriched in each entry was counted. Gene Ontology (GO) terms and KEGG pathways significantly enriched among the DEGs were identified by comparing against the whole-genome background. The results were visualized using scatter plots.

### RNA extraction and quantitative real-time PCR (qRT-PCR)

Total RNA was extracted from 2 mL of bacterial culture (OD₆₀₀ = 1.0) using Trizol reagent (INVITROGEN TRADING, Shanghai, China). Reverse transcription was performed according to the instructions of the reverse transcription kit (Vazyme, Nanjing, China). The sequences of qPCR primers are listed in Supplementary Table 1. qPCR was carried out using the SYBR qPCR Master Mix kit (Vazyme, Nanjing, China) on an Applied Biosystems QuantStudio 5 system (Thermo Fisher Scientific, Waltham, USA). The relative expression levels were calculated using the 2^–ΔΔCt^ method.

### Construction of deletion mutants

Using the *menB* (NP_416566.1) and *wza* (NP_416764.1) gene sequences retrieved from the NCBI database, primers for gene deletion in APEC94 and corresponding external verification primers were designed using Primer5 software (Version 5.0). All oligonucleotides were synthesized by Sangon Biotech (Shanghai, China). The specific primers used for gene knockout are listed in Supplementary Table 2. Using genomic DNA from the APEC94 strain as a template, the upstream and downstream homologous arms of *menB* and *wza*, as well as sgRNA fragments, were amplified by PCR. The purified homologous arm fragments and sgRNA fragments were then used as templates for overlap PCR assembly. The pTarget plasmid was digested with the restriction enzymes SpeⅠ and HindⅢ (BioScience, Shanghai, China), and the assembled fragment was ligated into the linearized pTarget vector. The pCas plasmid and the recombinant pTarget plasmid were successively electrotransformed into APEC94 cells. After recovery at 30 °C for 1 h, the transformants were plated on LB agar containing kanamycin and spectinomycin. Single colonies were picked and cultured in liquid LB medium, and successful integration was verified by PCR using external primers. Positive clones were inoculated into LB broth supplemented with kanamycin and ampicillin (Sangon Biotech, Shanghai, China), induced with 1 mM IPTG (Sigma-Aldrich, Shanghai, China), and cultured in a shaking incubator at 30 °C, 180 rpm for 12 h. The cultures were then plated on LB agar containing kanamycin and ampicillin. Individual colonies were selected and grown in liquid LB medium, and PCR was performed to confirm the elimination of the pTarget plasmid. Strains that had successfully lost the pTarget plasmid were inoculated into LB medium containing ampicillin and cultured at 42 °C with shaking at 180 rpm for 12 h to facilitate the curing of the pCas plasmid. The cultures were plated on ampicillin-containing LB plates, and single colonies were screened by PCR to verify the loss of the pCas plasmid.

## Statistical analysis

All experiments were repeated at least three times. Data are presented as mean ± standard deviation (SD). Comparisons between two groups were performed using Student’s t-test, while comparisons among multiple groups were conducted using one-way analysis of variance (ANOVA) followed by Dunnett’s post hoc test. P-values were calculated using GraphPad Prism (v9.0). A P-value < 0.05 was considered statistically significant.

## Results

### 4-OI inhibits growth and biofilm formation in APEC94

**Fig. 1:**
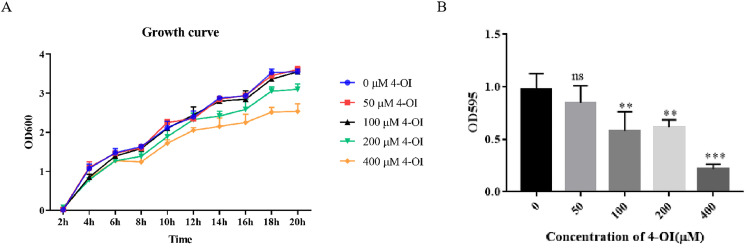
Growth curve and biofilm formation potential of APEC94 under the influence of 4-OI.

To investigate whether 4-OI could affect the growth and biofilm formation of APEC94, the bacteria were treated with varying concentrations of 4-OI. The results showed that the growth of APEC94 was significantly inhibited at 14, 16, 18, and 20 h after treatment with 400 µM 4-OI (Fig. 1A). Furthermore, biofilm formation was significantly inhibited following treatment with 100, 200, and 400 µM 4-OI (Fig. 1B). These results indicate that 4-OI can suppress both the growth and biofilm formation of APEC94.

(A) Bacterial growth curves show bacterial growth over time (x-axis) measured as OD₆₀₀ (y-axis), generated using Two-way ANOVA with Dunnett's post-hoc test comparing all groups to the 0 µM control at each time point. Treatment with 400 µM 4-OI significantly inhibited the growth of APEC94, with differences observed at 14, 16, 18, and 20 h (*p* < 0.0001). The blue curve indicates the group treated with an equivalent volume of PBS, the red curve represents 50 μM 4-OI, the black curve represents 100 μM 4-OI, the green curve represents 200 μM 4-OI, and the yellow curve represents 400 μM 4-OI. (B) Biofilm formation was assessed with 4-OI concentration plotted on the x-axis (0 μM denotes the control group treated with PBS) and OD₅₉₅ on the y-axis. Analysis with GraphPad Prism (version 9.0) showed that 100 µM, 200 µM, and 400 µM 4-OI significantly reduced biofilm formation in APEC94 (*p* < 0.01, *p* < 0.01, and *p* < 0.001, respectively).

### 4-OI enhances oxidative stress tolerance in APEC94

**Fig. 2:**
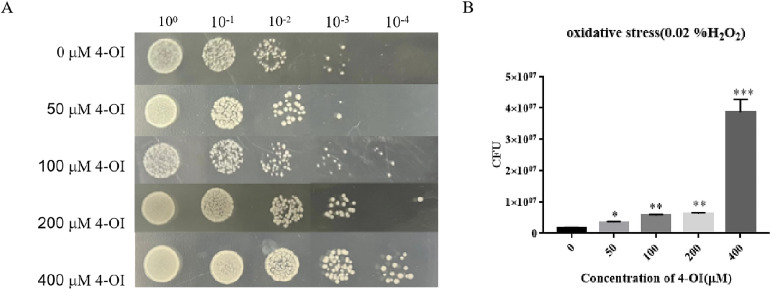
Oxidative stress measurement results of APEC94 under the influence of 4-OI.

To determine whether 4-OI affects the oxidative stress tolerance of APEC94, bacterial suspensions were serially diluted and treated with different concentrations of 4-OI under 0.02 % H₂O₂ stress. Colony counting results showed that at the 10⁻³ dilution, the number of APEC94 colonies treated with 100 μM, 200 μM, and 400 μM 4-OI was significantly higher than that in the untreated control group (Fig. 2A). Quantitative analysis further confirmed a significant increase in colony formation in 4-OI-treated groups compared to the untreated control (Fig. 2B). These results demonstrate that 4-OI enhances the oxidative stress tolerance of APEC94.

(A) The labels 100, 10⁻¹, 10⁻², 10⁻³, and 10⁻⁴ represent serial dilutions of the bacterial suspension in PBS. The y-axis shows the response to treatment with 0 to 400 µM 4-OI, where 0 µM 4-OI denotes the negative control group treated with an equivalent volume of PBS. (B) The x-axis indicates different concentrations of 4-OI, and the y-axis represents the CFU. Compared to the group without 4-OI, the addition of 50 µM, 100 µM, 200 µM, and 400 μM 4-OI significantly enhanced the CFU of APEC94 (*p* < 0.05, *p* < 0.01, *p* < 0.01, and *p* < 0.001, respectively).

### 4-OI diminishes acid-base stress tolerance in APEC94

**Fig. 3:**
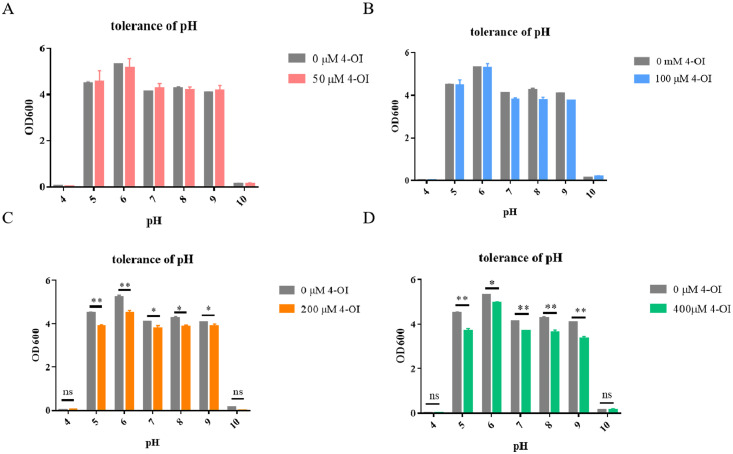
Acid-base tolerance test results of APEC94 under 4-OI treatment.

To investigate the effect of 4-OI on the acid-base tolerance of APEC94, bacteria were treated with different concentrations of 4-OI under varying pH conditions. The results showed that the growth of APEC94 was significantly impaired at pH values of 5, 6, 7, 8, and 9 following treatments with 200 μM and 400 μM 4-OI. These findings indicate that 4-OI decreases the acid-base stress tolerance of APEC94 (Fig. 3).

The x-axis represents different pH values, and the y-axis shows the OD₆₀₀ values (A–D). In all panels, "0 μM" denotes the control group treated with an equivalent volume of PBS. The bars in red, blue, yellow, and green correspond to treatment with 50 µM, 100 µM, 200 µM, and 400 µM 4-OI, respectively. The addition of 200 μM and 400 μM 4-OI markedly decreased the growth and viability of APEC94 at pH 5, 6, 7, 8, and 9 (*p* < 0.05).

### 4-OI diminishes osmotic stress tolerance in APEC94

**Fig. 4:**
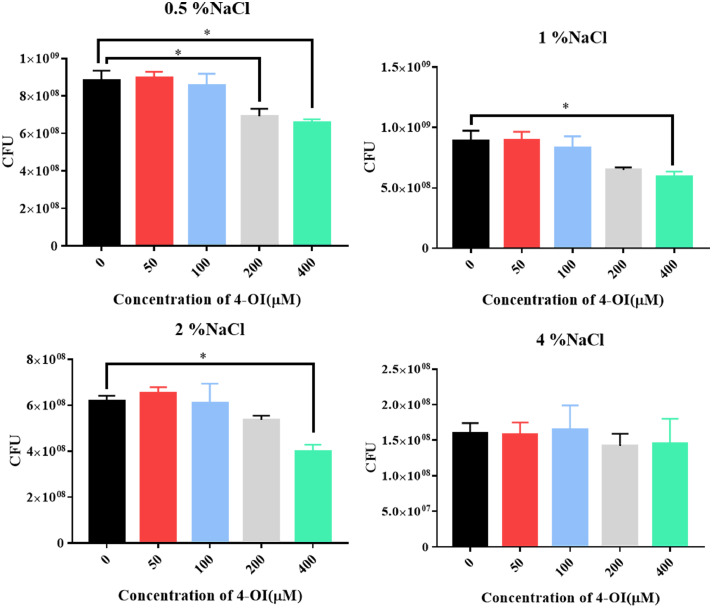
Osmo tolerance of APEC94 under 4-OI treatment.

To determine whether 4-OI affects the osmotic stress tolerance of APEC94 under salt-induced osmotic pressure, bacteria were treated with different concentrations of 4-OI in environments with varying NaCl concentrations. The results showed that 400 μM 4-OI significantly suppressed the growth of APEC94 under 0.5 %, 1 %, and 2 % NaCl conditions. Furthermore, 200 μM 4-OI also markedly reduced bacterial growth at 0.5 % NaCl. These results indicate that 4-OI diminishes the osmotic stress tolerance of APEC94 (Fig. 4).

The x-axis denotes different concentrations of 4-OI treatment, and the y-axis represents the number of CFU (A–D). Panel (A) corresponds to the 0.5 % NaCl environment, (B) to 1 % NaCl, (C) to 2 % NaCl, and (D) to 4 % NaCl. Under 0.5 %, 1 %, and 2 % NaCl conditions, 400 μM 4-OI markedly suppressed the growth and viability of APEC94 (*p < 0.05*). Additionally, in the 0.5 % NaCl environment, 200 μM 4-OI also resulted in a significant reduction of bacterial growth and survival (*p* < 0.05).

### Transcriptomic profiling reveals 4-OI-induced differential gene expression in APEC94

**Fig. 5:**
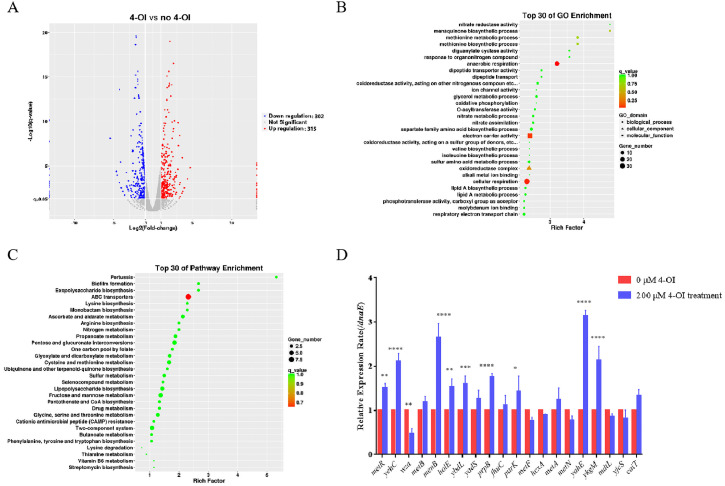
Transcriptomic analysis of the effects of exogenous 4-OI addition on APEC94 gene expression and RT-qPCR validation of differentially expressed genes.

To further elucidate the effect of 4-OI on the transcriptional profile of APEC94 and identify its potential target genes, APEC94 was treated with 200 μM 4-OI and subjected to transcriptomic sequencing analysis. Using a threshold of fold change ≥ 1.5 and a p-value < 0.05, we identified a total of 617 differentially expressed genes (DEGs), among which 315 were up-regulated and 302 were down-regulated (Fig. 5A). GO enrichment analysis of the significant DEGs revealed that the major functional terms enriched under 4-OI treatment were anaerobic respiration, electron carrier activity, and cellular respiration (Fig. 5B). KEGG pathway enrichment analysis indicated that the ABC transporter system was among the most significantly enriched pathways. A scatter plot visualizes the top 30 enriched GO terms (Fig. 5C). To further validate the impact of 4-OI on APEC94 biological functions, 20 differentially expressed genes were selected for RT–qPCR validation. *MetR, yehC, metB, menB, holE, ybdL, yadS, prpB, purK, metA, yahE, ykgM*, and *cait* were up-regulated, while *wza, fhuC, metF, hcxA, metN, mdtL*, and *yjcS* were down-regulated. The expression trends observed in RT–qPCR were consistent with the transcriptomic sequencing results (Fig. 5D). These findings confirm the reliability of the transcriptomic data and suggest that 4-OI modulates biological functions in APEC94, potentially by targeting *menB* (encoding a CoA synthase) and *wza* (encoding an outer membrane polysaccharide export protein), both of which showed substantial changes in expression.

(A). A volcano plot was generated with Log₂ (Fold change) on the x-axis and -Log₁₀(q-value) on the y-axis to visualize differentially expressed genes. Blue points represent the 4-OI treatment group, while red points indicate the PBS-treated negative control group. (B) GO enrichment analysis of differentially expressed genes was performed using GOseq. The numbers of differentially expressed genes associated with biological processes, cellular components, and molecular functions were summarized and presented in a GO enrichment scatter plot. (C) A KEGG enrichment scatter plot of differentially expressed genes was generated, displaying the top 30 GO entries ranked by enrichment factor. (D) The x-axis represents the differentially expressed genes detected, and the y-axis shows the relative gene expression levels. Red bars denote the negative control group treated with an equivalent volume of PBS, and blue bars represent the group treated with 200 µM 4-OI.

### *menB* and *wza* are required for 4-OI-mediated inhibition of biofilm formation

**Fig. 6:**
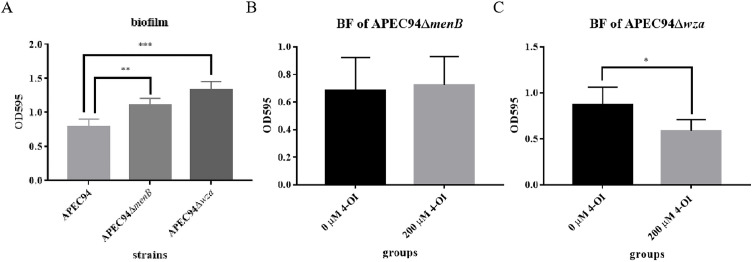
Assessment of biofilm formation capabilities in APEC94ΔmenB and APEC94Δwza with and without 4-OI supplementation.

To investigate the roles of the *menB* and *wza* genes in the interaction between APEC94 and 4-OI, deletion mutants of *menB* and *wza* were constructed (Fig. S1), and the effect of 4-OI on biofilm formation in these mutant strains was assessed. The results showed that biofilm formation was significantly enhanced in both APEC94ΔmenB and APEC94Δwza mutants relative to the wild-type strain (Fig. 6A). After the addition of 200 μM 4-OI, biofilm formation in the APEC94ΔmenB mutant showed no significant change (Fig. 6B). In contrast, a substantial reduction in biofilm formation was observed in the APEC94Δwza mutant treated with 200 μM 4-OI (Fig. 6C). These findings indicate that both *menB* and *wza* genes suppress biofilm formation in APEC94 and that 4-OI further inhibits biofilm formation by targeting these genes.

The x-axis in each panel represents different treatment groups: APEC94, APEC94ΔmenB, and APEC94Δwza in (A); 0 μM and 200 μM 4-OI treatments in (B) and (C). The y-axis indicates the OD₅₉₅ values, reflecting biofilm formation. (A) Deletion of *menB* or *wza* significantly promoted biofilm formation in APEC94 (*p* < 0.01 and *p* < 0.001, respectively). (B) Biofilm formation in the APEC94ΔmenB mutant showed no significant change after 200 μM 4-OI treatment (*p* > 0.05). (C) A significant reduction in biofilm formation was observed in the APEC94Δwza mutant following treatment with 200 μM 4-OI (*p* < 0.05).

### 4-OI alters oxidative stress tolerance in APEC94ΔmenB and APEC94Δwza mutant strains

**Fig. 7:**
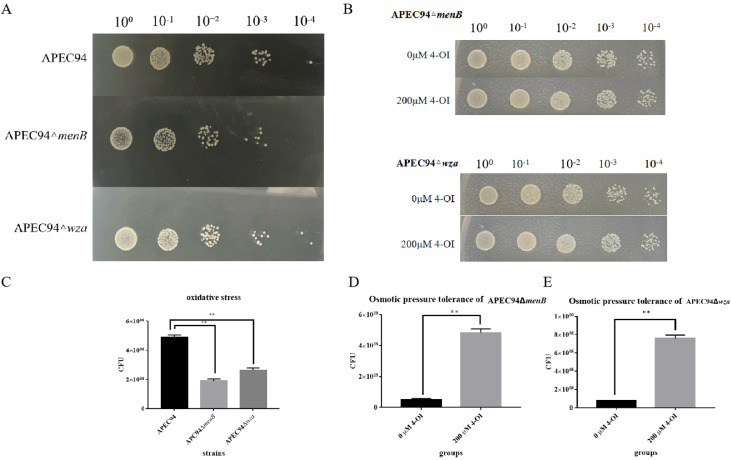
Effect of 4-OI Treatment on Oxidative Stress Tolerance in APEC94ΔmenB and APEC94Δwza Mutants.

To further examine the roles of the *menB* and *wza* genes in mediating the effect of 4-OI on the oxidative stress tolerance of APEC94, bacterial cultures were serially diluted and treated with 4-OI. The results showed that, compared to the wild-type strain, the deletion of *menB* or *wza* led to a significant reduction in colony growth under oxidative stress conditions (Fig. 7A). Colony counting assays further confirmed that oxidative stress tolerance was significantly decreased in both the APEC94ΔmenB and APEC94Δwza mutants relative to the wild-type APEC94 (Fig. 7C). At the 10⁻⁴ dilution, the addition of 200 μM 4-OI resulted in a higher number of colonies in both the APEC94ΔmenB and APEC94Δwza mutants compared to the untreated groups (Fig. 7B). Consistent with this, oxidative stress tolerance was significantly enhanced in both mutant strains following 4-OI treatment (Fig. 7D and E). These findings demonstrate that both *menB* and *wza* genes contribute to the growth of APEC94, and that 4-OI enhances the antioxidant stress tolerance of APEC94 by acting through these genes.

(A). The x-axis labels 10⁰, 10⁻¹, 10⁻², 10⁻³, and 10⁻⁴ indicate serial dilutions of the bacterial suspension in PBS. The y-axis represents the treatment groups: APEC94, APEC94ΔmenB, and APEC94Δwza. (B) The x-axis again denotes serial dilutions (10⁰ to 10⁻⁴) in PBS. The y-axis represents the treatment groups: 0 μM 4-OI, 200 μM 4-OI, APEC94ΔmenB, and APEC94Δwza. (C) The x-axis shows the treatment groups: APEC94, APEC94ΔmenB, and APEC94Δwza. The y-axis indicates the CFU count. The antioxidant stress capacity was markedly diminished in both APEC94ΔmenB and APEC94Δwza compared to the wild-type APEC94 (*p* < 0.01). (D) The x-axis compares the 0 μM 4-OI treatment group (negative control treated with PBS) and the 200 μM 4-OI treatment group. The y-axis shows the CFU count. The results demonstrate a significant increase in CFU for APEC94ΔmenB following treatment with 200 μM 4-OI (*p* < 0.01). (E) The x-axis compares the 0 μM 4-OI group (PBS-treated negative control) and the 200 μM 4-OI group. The y-axis indicates the CFU count. A significant increase in CFU was observed in APEC94Δwza after treatment with 200 μM 4-OI (*p* < 0.01).

### The roles of *menB* and *wza* in acid-base and osmotic stress tolerance

**Fig. 8:**
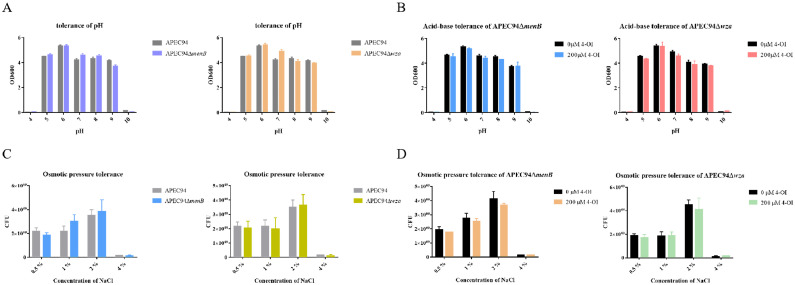
Evaluation of Acid-Base Tolerance in APEC94ΔmenB and APEC94Δwza Mutants with and without 4-OI Supplementation.

To further investigate the roles of *menB* and *wza* genes in mediating the effects of 4-OI on acid-base stress tolerance and osmotic stress tolerance in APEC94, bacterial strains were treated with 4-OI under varying pH and osmotic conditions. The results showed that, compared to the wild-type strain, the deletion mutants exhibited no significant difference in acid-base stress tolerance (Fig. 8A). Similarly, after treatment with 200 μM 4-OI, the acid-base stress tolerance of the mutant strains showed no notable change (Fig. 8B). Likewise, no significant difference in osmotic stress tolerance was observed between the gene deletion mutants and the wild-type strain (Fig. 8C). The addition of 200 μM 4-OI did not alter osmotic stress tolerance in the mutants (Fig. 8D). These results indicate that the deletion of *menB* or *wza* does not significantly alter acid-base or osmotic stress tolerance in APEC94. The finding that 4-OI treatment also had no significant effect in the mutant backgrounds suggests that the mechanisms by which 4-OI diminishes acid-base and osmotic stress tolerance are complex and may involve targets beyond *menB* and *wza*.

(A) The x-axis indicates different pH values, and the y-axis represents the OD₆₀₀ values. The black bars represent the APEC94 group, the blue bars represent the APEC94ΔmenB mutant, and the orange bars represent the APEC94Δwza mutant. (B) The x-axis shows different pH values, and the y-axis displays the OD₆₀₀ values. The black bars correspond to the 0 μM 4-OI treatment group, the blue bars indicate the APEC94ΔmenB mutant treated with 200 μM 4-OI, and the red bars indicate the APEC94Δwza mutant treated with 200 μM 4-OI. (C) The x-axis denotes different NaCl concentrations, and the y-axis shows the CFU count. The gray bars represent the APEC94 group, the blue bars represent the APEC94ΔmenB mutant, and the yellow bars represent the APEC94Δwza mutant. (D) The x-axis represents different NaCl concentrations, and the y-axis indicates the CFU count. The black bars correspond to the 0 μM 4-OI treatment group, the orange bars represent the APEC94ΔmenB mutant treated with 200 μM 4-OI, and the blue bars represent the APEC94Δwza mutant treated with 200 μM 4-OI.

## Discussion

APEC is a small, rod-shaped, Gram-negative bacterium that possesses flagella or fimbriae and is enclosed in a lipopolysaccharide (LPS) outer membrane. It is capable of causing various localized and systemic diseases in poultry (Nawaz, et al., 2024). APEC often exists as a commensal member of the avian intestinal microbiome but can disseminate upon inhalation or entry into non-intestinal sites, leading to conditions such as respiratory disease (airsacculitis), sepsis (pericarditis and perihepatitis), and other disorders, including salpingitis, cellulitis, and joint infections (de Oliveira, et al., 2020; Poulsen, et al., 2020). Certain APEC outbreak events in production facilities can result in mortality rates as high as 20 %. Additionally, the combined losses from reduced weight gain and downgrading or condemnation of infected carcasses impose significant economic burdens on the global poultry industry (Ghunaim, et al., 2014; Kim, et al., 2020). Therefore, preventing and controlling the occurrence and transmission of avian colibacillosis are of considerable importance (Kabiswa, et al., 2018). However, the emergence of antibiotic-resistant APEC strains poses a major global health threat, compromising both animal health and food safety (Thomrongsuwannakij, et al., 2020). Consequently, alternative strategies, including vaccines, probiotics, and bacteriophages, have attracted considerable attention as replacements for antibiotics (Joseph, et al., 2023).

Recently, itaconate, an intermediate metabolite of the TCA cycle, has garnered growing scientific interest (Shi, et al., 2022). Itaconate is a highly polar, α, β-unsaturated dicarboxylic acid featuring a double bond and two carboxyl groups. Owing to its chemical properties, transmembrane transport of exogenous itaconate into the cytoplasm is likely restricted. To address this issue, researchers have synthesized various derivatives, such as dimethyl itaconate (DI) and 4-ethyl itaconate (4-EI), to elucidate their functional roles (Shi, Zhou, Wei, Mo, Li and Lv, 2022). Mills et al. developed 4-octyl itaconate (4-OI), an ester derivative with a distal ester group relative to the alkene, resulting in reduced thiol reactivity (Mills, et al., 2018). It has been found that itaconate can effectively inhibit the growth of various bacteria, including *Salmonella, Mycobacterium tuberculosis*, and *Legionella pneumophila* under glucose-deficient conditions by suppressing the activity of ICL (Li, et al., 2023; Michelucci, et al., 2013; Naujoks, et al., 2016). Furthermore, itaconate can inhibit other bacterial enzymes beyond ICL, such as propionyl-CoA decarboxylase (in *Rhodospirillum rubrum*) and methylmalonyl-CoA mutase (in *M. tuberculosis*) (Berg, et al., 2002). The antibacterial activity of itaconate is significantly enhanced in acidic environments, as low pH promotes its "proton shuttle" effect, which disrupts bacterial cytoplasmic pH and exerts toxicity (Duncan, et al., 2021).

However, the effect of itaconate on APEC remains unreported. This study investigated the impact of 4-OI on the biological characteristics of APEC. The results demonstrated that exogenous addition of 4-OI significantly inhibited bacterial growth and biofilm formation. Transcriptomic analysis revealed that 4-OI markedly altered the transcriptional levels of the *wza* and *menB* genes. The *wza* gene encodes an outer membrane polysaccharide export protein involved in the secretion and assembly of capsular polysaccharides. In APEC, the capsule is a key virulence factor that enables the bacteria to evade phagocytosis by the host immune system and enhances their survival within the host (Johnson, et al., 2006). Downregulation of *wza* impairs capsule formation, increasing bacterial surface hydrophilicity and potentially reducing adhesion, thereby inhibiting biofilm formation and increasing susceptibility to environmental stresses. The *menB* gene encodes a menaquinone synthase, which participates in the biosynthesis of vitamin K₂ (menaquinone). Vitamin K₂ acts as a vital cofactor in the bacterial respiratory chain, facilitating electron transfer, thereby influencing energy metabolism and oxidative stress resistance. In APEC, *menB* may indirectly support virulence expression by sustaining metabolic activity under host hypoxic conditions, such as those at infection sites (Quinn, et al., 2014). As menaquinone is involved in the electron transport chain, its perturbation by 4-OI could alter the redox state of the cell, potentially explaining the complex effects on oxidative stress tolerance. Therefore, based on the transcriptomic sequencing results, we hypothesize that 4-OI influences the biological functions of APEC94 by acting on the *wza* and *menB* genes. To further validate this hypothesis, we constructed *wza* and *menB* gene knockout mutants of APEC. We found that the reduction in acid-base and osmotic stress tolerance induced by 4-OI was not observed in the APEC94ΔmenB and APEC94Δwza mutants, indicating that the presence of these genes is required for 4-OI to exert these particular inhibitory effects, although the precise mechanism may involve a more complex regulatory network beyond direct targeting.

While the observed enhancement of H₂O₂-induced oxidative stress tolerance by 4-OI seems counterintuitive for an antibacterial agent, it is crucial to consider the net effect on bacterial fitness (Zenin, et al., 2022). The potent inhibition of growth, biofilm formation, and tolerance to acid-base and osmotic stresses likely represents a more significant impairment to APEC's overall pathogenicity and ability to establish an infection in the host. The complex host environment presents multiple simultaneous stresses, and the net antibacterial effect of 4-OI, resulting from the combination of all these phenotypic alterations, is ultimately inhibitory (Cotten and Davis, 2023).

The relationship between *wza, menB*, and the observed phenotypes appears complex. The deletion of *wza* likely disrupts capsular polysaccharide export, increasing bacterial surface hydrophilicity and potentially altering cell-surface interactions in a way that, paradoxically, enhanced biofilm formation in our assay (Fazeli-Nasab, et al., 2022). Conversely, 4-OI-mediated downregulation of *wza* in the wild-type strain may achieve a subtler modulation that ultimately inhibits proper biofilm maturation. Similarly, while *menB* deletion cripples the electron transport chain, reducing energy metabolism and antioxidant capacity, 4-OI-induced upregulation of *menB* may enhance these processes. This indicates that *wza* and *menB* operate in distinct yet interconnected networks regulating biofilm formation and oxidative stress response, and 4-OI exerts its effects by differentially modulating these key nodes (Liao, et al., 2019).

Our study elucidates the effects of 4-OI on the biological characteristics of APEC, thereby laying a theoretical foundation for understanding its mechanism of action. Moreover, in the context of increasing serotype diversity and antimicrobial resistance in APEC, this research provides new insights for the development of novel antibiotic-alternative strategies to prevent and control APEC infections. A key remaining question is how 4-OI regulates the transcription of *menB* and *wza*. As a derivative of an electrophilic metabolite, 4-OI directly modifies a bacterial transcriptional regulator through alkylation, analogous to its known effect on KEAP1 in mammalian cells. Identifying this putative regulator represents a crucial next step in fully elucidating the mechanism of 4-OI's action in bacteria. A limitation of this study is the absence of double-gene knockout (APEC94ΔmenB and APEC94Δwza) and genetic complementation strains. The construction and phenotyping of these strains are essential future steps to conclusively delineate the individual and synergistic contributions of *menB* and *wza* to the multifaceted effects of 4-OI.

## Conclusion

Our findings indicate that both *menB* and *wza* genes are involved in suppressing biofilm formation in APEC94 under baseline conditions. Furthermore, 4-OI can inhibit biofilm formation through additional mechanisms that are independent of *wza*, as evidenced by its effect in the APEC94Δwza mutant. A comprehensive understanding will require future studies with double-knockout and complementary strains.

## Data availability

The data sets generated for this study are available at Doi: 10.17632/swkkk7rcdc.1.

## Availability of data and materials

All data generated or analyzed during this study are included in this published article.

## CRediT authorship contribution statement

**Chuanyan Che:** Writing – original draft. **Xiuqi Fan:** Writing – original draft. **Haiyang Wang:** Writing – original draft. **Zhihao Wang:** Writing – original draft. **Yinli Bao:** Funding acquisition. **Wei Jiang:** Funding acquisition. **Saqib Nawaz:** Writing – original draft. **Huifang Yin:** Funding acquisition. **Xiangan Han:** Funding acquisition.

## Disclosures

The authors declare that they have no competing interests.

## Appendix Supplementary materials

Image, image 1

Image, application 2

Supplementary Figure 1 (Figure S1). PCR detection of external verification primers for gene deletion mutants. Image, application 3
