# Supplementary figures and images for: 4-octyl itaconate modulates virulence-associated phenotypes and oxidative stress resistance in avian pathogenic Escherichia coli by targeting menB and wza
Source: Poult Sci. 2025 Dec 6;105(2):106202. doi: 10.1016/j.psj.2025.106202 (PMC12756159; doi:10.1016/j.psj.2025.106202)

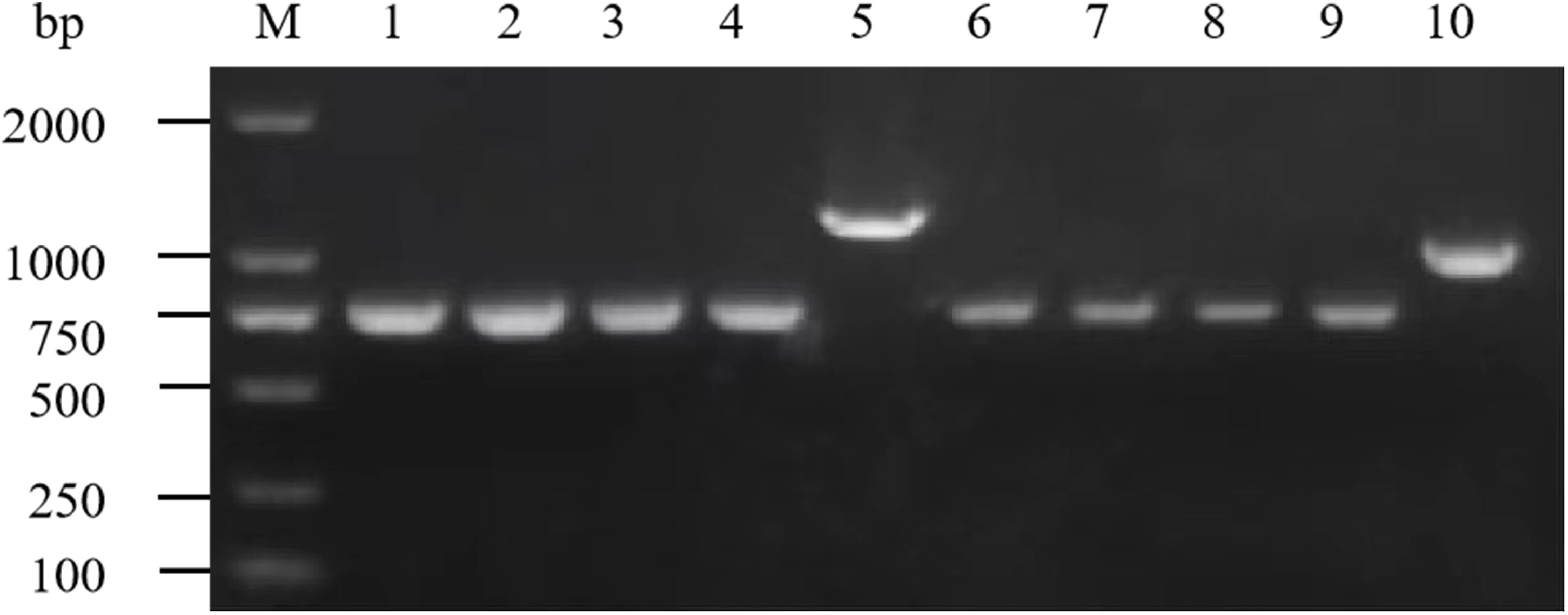

Supplement: Supplementary file 1 [file mmc1.jpg]
