# Supplementary material for: 4-octyl itaconate modulates virulence-associated phenotypes and oxidative stress resistance in avian pathogenic Escherichia coli by targeting menB and wza
Source: Poult Sci. 2025 Dec 6;105(2):106202. doi: 10.1016/j.psj.2025.106202 (PMC12756159; doi:10.1016/j.psj.2025.106202)
Supplement: Supplementary file 2 [file mmc2.docx]

**Supplementary Table 1**

| **Primer name** | **Primer sequence (5’-3’)** |
| --- | --- |
| RT-met-F | TGAAACGCAAAGTGGATGCC |
| RT-met-R | AAACGCAGGTAGCTTTCGAC |
| RT-hcxA-F | TGCACCGCAACAATATCTGC |
| RT-hcxA-R | TAACAAGACGTCGCGAATGG |
| RT-metA-F | TTACTGGCCGCAGATCAAAC |
| RT-metA-R | GTTTTTCGGTGCGAGTTTGC |
| RT-ybdL-F | TTTTCACAACAGGGCCATGC |
| RT-ybdL-R | AATGAAGAAACCGCCACTGC |
| RT-yehC-F | TGCACTGAAGTTTGCGATGC |
| RT-yehC-R | CCATTGCCACTGCGATTTACC |
| RT-metR-F | AACGCGAATTACACCGGAAG |
| RT-metR-R | TGAAGAAAATGCCGCCAGAC |
| RT-holE-F | TGAAGCGGTTGAACGTGAAC |
| RT-holE-R | AGATTGACCGAAGCCAAACG |
| RT-purK-F | GGTGCCTTTTCAACAAAGCG |
| RT-purK-R | ACACATCGCGGTTCACAAAG |
| RT-fhuC-F | AACGCCTGCGGAAATTATGC |
| RT-fhuC-R | ATGCGGCAAAATACCCATCG |
| RT-metN-F | TTTTTGGCAACGTGGCTCTG |
| RT-metN-R | ATGACAGCAATTCCGTCACG |
| RT-prpB-F | TTACCACCGACGAATTACGC |
| RT-prpB-R | TGTCGATGACGCTTTTCTGC |
| RT-yadS-F | TTGCAATGGTCACCAGCATG |
| RT-yadS-R | ACCGGCAACATCCATTTTGG |
| RT-rcsA-F | TGCGGCATTTAACAGTGTGG |
| RT-rcsA-R | TCAAGCTGCAAACGAACGTC |
| RT-yahE-F | TTTATCTCGGTCTGCAACGC |
| RT-yahE-R | ATATTTTCCCGGCAGCGTTG |
| RT-ykgM-F | ATTGAGCTGGATGGCGTAAC |
| RT-ykgM-R | ATGCCACTGTTCTCAGCTTC |
| RT-mdtI-F | ATCGTGCTGGAAATCGTTGC |
| RT-mdtI-R | TGAGAAAGCGCACTAAAGGC |

**Table continued**

| **Primer** | **Primer sequence (5’-3’)** |
| --- | --- |
| RT-caiT-F | AACACGCCAAAGGGTTGTTC |
| RT-caiT-R | ATGCGGAATGCCAAACAACC |
| RT-yjcS-F | AGAAGCGAACAAGCAAGGTG |
| RT-yjcS-R | TTCGCTTGCAGATTCTTCGC |
| RT-menB-F | ACATCGGCGTGATCATTCTG |
| RT-menB-R | AATCACCACGCACTTTCTGG |
| RT-wza-F | AGCGGCATCTTTGTCATTCG |
| RT-wza-R | TAAGGTTGCAGGCGGAATTC |
