# Supplementary material for: 4-octyl itaconate modulates virulence-associated phenotypes and oxidative stress resistance in avian pathogenic Escherichia coli by targeting menB and wza
Source: Poult Sci. 2025 Dec 6;105(2):106202. doi: 10.1016/j.psj.2025.106202 (PMC12756159; doi:10.1016/j.psj.2025.106202)
Supplement: Supplementary file 3 — Supplementary Figure 1 (Figure S1). PCR detection of external verification primers for gene deletion mutants. [file mmc3.docx]

**Supplementary Table 2:**

| **Primers name** | **Primer sequence (5’-3’)** | **Length/bp** |
| --- | --- | --- |
| menB-UF  menB-UR | GCTTTTTTTGATGATTTATCCTGATGAAGC  ACTGACGGCAATCATCTTTATAGCCGCCGT | 300 |
| menB-DF  menB-DR | TAAAGATGATTGCCGTCAGTACGACGCAAA  CCAAGCTTTTACGGATTCCGTTTGAATT | 300 |
| menB-sgRNA-F  menB-sgRNA-R | GGACTAGTGCGGCAGATAATGCCATCTTGTTTTAGAGCTAGAAATAGC  GATAAATCATCAAAAAAAGACTCGG | 106 |
| menB-out-F  menB-out-R | TGGTGAACGTGACAGCAAATTCC  CCAAGCTTTTACGGATTCCGTTTGAATT | 1014 |
| wza-UF  wza-UR | GCTTTTTTTGGTAACCTTGATAAGCGGTTGCAC  GCTCTGTTTCAAAACATAGTGCCGTCAGGCTG | 340 |
| wza-DF  wza-DR | ACTATGTTTTGAAACAGAGCACCCTGAAAATGG  CCAAGCTTTTACCAGTTATGAATGTCGCTGGC | 349 |
| wza-sgRNA-F  wza-sgRNA-R | GGACTAGTGCCTGACCGACACCGCTGACGTTTTAGAGCTAGAAATAGC  TCAAGGTTACCAAAAAAAGACTCGG | 103 |
| wza-out-F  wza-out-R | ATAAGCGGTTGCACAGTACTTCC  CCAAGCTTTTACCAGTTATGAATGTCGCTGGC | 1089 |
| pTarget-F  pTarget-R | AGCGAGGAAGCGGAAGAGCG  CAAGATAGCCAGATCAATGT | 800 |
